# Identification of Angiogenesis Rich-Viable Myocardium using RGD Dimer based SPECT after Myocardial Infarction

**DOI:** 10.1038/srep27520

**Published:** 2016-06-10

**Authors:** Min Su Lee, Hyun Soo Park, Byung Chul Lee, Jae Ho Jung, Jung Sun Yoo, Sang Eun Kim

**Affiliations:** 1 Department of Nuclear Medicine, Seoul National University Bundang Hospital, Seoul National University College of Medicine, Seoul, Republic of Korea; 2 Smart Humanity Convergence Center, Program in Biomedical Radiation Sciences, Department of Transdisciplinary Studies, Graduate School of Convergence Science and Technology, Seoul National University, Seoul, Republic of Korea; 3 Center for Nanomolecular Imaging and Innovative Drug Development, Advanced Institutes of Convergence Technology, Suwon, Republic of Korea

## Abstract

Cardiac healing after myocardial ischemia is a complex biological process. Advances in understanding of wound healing response have paved the way for clinical testing of novel molecular imaging to improve clinical outcomes. A key factor for assessing myocardial viability after ischemic injury is the evaluation of angiogenesis accompanying increased expression of integrin α_v_β_3_. Here, we describe the capability of an α_v_β_3_ integrin-targeting SPECT agent, ^99m^Tc-IDA-D-[c(RGDfK)]_2_, for identification of ischemic but viable myocardium, i.e., hibernating myocardium which is crucial to predict functional recovery after revascularization, the standard care of cardiovascular medicine. *In vivo* SPECT imaging of rat models with transient coronary occlusion showed significantly high uptake of ^99m^Tc-IDA-D-[c(RGDfK)]_2_ in the ischemic region. Comparative measurements with ^201^Tl SPECT and ^18^F-FDG PET, then, proved that such prominent uptake of ^99m^Tc-IDA-D-[c(RGDfK)]_2_ exactly matched the hallmark of hibernation, i.e., the perfusion-metabolism mismatch pattern. The uptake of ^99m^Tc-IDA-D-[c(RGDfK)]_2_ was non-inferior to that of ^18^F-FDG, confirmed by time-course variation analysis. Immunohistochemical characterization revealed that an intense signal of ^99m^Tc-IDA-D-[c(RGDfK)]_2_ corresponded to the vibrant angiogenic events with elevated expression of α_v_β_3_ integrin. Together, these results establish that ^99m^Tc-IDA-D-[c(RGDfK)]_2_ SPECT can serve as a sensitive clinical measure for myocardial salvage to identify the patients who might benefit most from revascularization.

Cardiovascular diseases (CVD), such as myocardial infarction (MI), are the leading cause of morbidity and mortality worldwide, causing 31% of all global deaths^1^ and are associated with the steeply growing cost of health care. MI is mainly caused by a blockage of the coronary blood supply to the myocardium and results in irreversible damage, including myocardial loss, ventricular remodeling (i.e., adverse structural alterations due to poor infarct healing), cardiac dysfunction, and heart failure. Timely restoration of blood flow to the ischemic myocardium (reperfusion) has been the standard care of the patients presenting early after symptom onset. Reperfusion or revascularization therapy is highly efficient in limiting infarct size, improving long-term myocardial function, changing the healing pattern of the infarcted zone, and more importantly, reducing mortality. To get success of such therapy, it is crucial to identify hibernating (i.e., dysfunctional but viable) myocardium after ischemic injury with noninvasive means because revascularization has the potential to restore contractile function of hibernation but not scar (i.e., irreversible loss of myocardium).

Traditionally, monitoring of CVD was based on techniques that measure changes in blood flow and cellular metabolism. Radiotracers such as thallium-201 (^201^Tl) and technetium-99 m (^99m^Tc) sestamibi are taken up by cardiomyocytes and hence their homogeneous uptake reflects the combination of normal myocytes’ distribution and myocardial perfusion while a defect signal indicates an area with loss of viable myocardium^2^,^3^. Although myocardial perfusion imaging has been a major tool in the evaluation of CVD with a vast evidence base in over 100,000 patients since 1970’s, limitations are becoming apparent since single photon emission computed tomography (SPECT) imaging with ^201^Tl or ^99m^Tc sestamibi cannot provide any molecular and pathophysiological insight on the defect region and utilize relatively high ionizing radiation. ^18^F-fluoro-2-deoxy-D-glucose (^18^F-FDG) positron emission tomography (PET) is another gold standard to measure myocardial viability more sensitively. The uptake and retention of ^18^F-FDG reflects the activity of the various glucose transporters and hexokinase-mediated phosphorylation. In the setting of ischemic heart failure, viable myocardium often exhibits a shift in substrate utilization from aerobic (free fatty acids) to anaerobic (glucose) metabolism, thus, ^18^F-FDG imaging provides an *in vivo* assessment tool for glycolytic activity of the ischemic myocardium and can be used to evaluate myocardial viability. A combination of perfusion and glucose metabolism imaging enables classification of myocardium, i.e., fibrous scar, when there is a decrease both in perfusion and metabolism; viable myocardial hibernation, when a perfusion/metabolic mismatch occurs; and normal tissue, when myocardial perfusion and metabolism were preserved^4^. Despite such well-established value for clinical assessment, this provides only little insight into the underlying biological processes after initiation of MI which makes it difficult to predict future cardiovascular events and assess individual efficacy of reperfusion therapy. To distinguish ischemically compromised but viable “hibernating myocardium” before manifestation of adverse remodeling, specifically targeted imaging technique of evaluating critical molecular processes is needed.

Myocardial ischemia results in hypoperfusion and tissue hypoxia, leading to the stimulation of angiogenesis, i.e., formation of new capillaries from existing microvessels. Accordingly, angiogenesis is considered as an important component of infarct healing which can be a key biomarker to delineate viable myocardium early after MI. In addition, it has been a target of molecular therapies to direct myocardial repair with several clinical trials^5^,^6^,^7^. Integrin α_v_β_3_, i.e., a cell membrane glycoprotein receptor that is highly expressed on endothelial cells during angiogenesis, has been identified as a favorable target for imaging angiogenesis and thus has attracted great interest in the field of MI staging^8^,^9^. Cumulative studies have revealed that its expression is up-regulated within the first few weeks after ischemic myocardial injury in the infarcted and border zone regions as part of the early infarct healing process^10^,^11^,^12^. Most notably, a recent study showed that a strong early integrin imaging signal from the ischemic region in rats was associated with less ventricular remodeling in subsequent weeks, suggesting that integrin expression is a potential biomarker of cardiac repair^13^. Imaging studies mainly focused on cyclic arginyl-glycyl-aspartic acid (RGD) peptide, a potent inhibitor of α_v_β_3_ integrin, which was radiolabeled for SPECT or PET imaging of experimental MI models^10^,^14^,^15^,^16^,^17^,^18^,^19^,^20^. However, as yet none have shown comparison with combined use of perfusion and metabolic imaging, i.e., the most established clinical protocols. In addition, most trials have not carefully considered pathological advance and therapeutic process of MI by using simple permanent coronary artery ligation model, revealing limitations to return to the bedside for clinical testing.

We recently developed a new radiotracer, ^99m^Tc-labeled RGD peptide (^99m^Tc-IDA-D-[c(RGDfK)]_2_) and successfully applied for diagnostic SPECT imaging of glioblastoma^21^ and atherosclerosis^22^. Considering high integrin-binding affinity, specific *in vivo* targeting, and desirable pharmacokinetic properties as shown in the previous studies^21^,^22^, the developed RGD dimer agent is expected to be suitable to pinpoint hibernating myocardium which is clinically characterized by perfusion defect and enhanced FDG uptake. To prove such utility of ^99m^Tc-IDA-D-[c(RGDfK)]_2_ SPECT imaging approach, we particularly focused on *in vivo* imaging of transient coronary occlusion model to mimic reversible myocardial infarction and reperfusion in the clinical setting. Comparative measurements with ^201^Tl SPECT and ^18^F-FDG PET, then, showed that focal uptake of ^99m^Tc-IDA-D-[c(RGDfK)]_2_ matched the hallmark of hibernation, i.e., the perfusion-metabolism mismatch pattern. Together, we demonstrate a molecular imaging strategy that uses α_v_β_3_ integrin-targeted probe ^99m^Tc-IDA-D-[c(RGDfK)]_2_ with SPECT to assess myocardial viability after ischemic injury to identify the patients who might benefit most from revascularization.

## Results

### Generation of MI/reperfusion Model and Work Flow of Molecular Imaging

**Figure 1:**
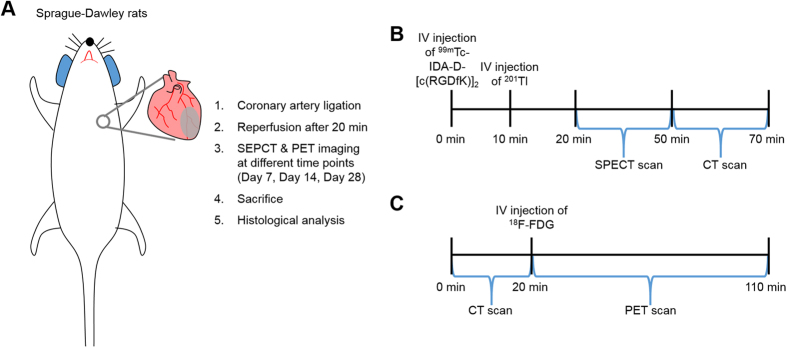
Schematic work flow of model generation and molecular imaging. (**A**) Diagram of whole experimental procedure. The coronary artery was ligated to create myocardial infarction (MI) in Sprague-Dawley (SD) rats, followed by reperfusion after 20 minutes. Single photon emission computed tomography (SPECT) and positron emission tomography (PET) imaging were performed 7, 14, and 28 days after MI. After *in vivo* imaging, animals were sacrificed for histological analyses. (**B**) SPECT scans were obtained for 30 minutes at 10 min and 20 min post-injection of ^201^Tl and ^99m^Tc-IDA-D-[c(RGDfK)]_2_, respectively, immediately followed by 20-min computed tomography (CT) scans. (**C**) ^18^F-FDG PET/CT was conducted with the identical animal at the same day of SPECT/CT imaging. After CT data acquisition for 20 minutes, the PET images were obtained for 90 minutes with intravenous injection of ^18^F-FDG.

To investigate the ability of α_v_β_3_ integrin-targeted probe to detect hibernating myocardium which is crucially important in clinical decision-making, we established clinically relevant models of myocardial infarction (MI)/reperfusion by transient coronary occlusion of rats (n = 4). Figure 1 shows schematic work flow of model generation and molecular imaging. The anterior descending branch of the left coronary artery was ligated to create MI in rats, followed by reperfusion after 20 minutes to induce dysfunctional but viable (hibernating) myocardium (Fig. 1A). After 7, 14, and 28 days, animals were subjected to single photon emission computed tomography (SPECT) and positron emission tomography (PET) imaging, and sacrificed for histological analyses (Fig. 1A). We performed three different tracers’ imaging, i.e, ^99m^Tc-IDA-D-[c(RGDfK)]_2_ SPECT, ^201^Tl SPECT, and ^18^F-FDG PET at different time points (7, 14, and 28 days post-injury) on each single animal repetitively. This made it possible to minimize the number of animals used and improve reliability of comparative imaging data by testing each tracer on the exactly same state of myocardium. Specifically, SPECT scans were performed for 30 minutes after serial injection of ^99m^Tc-IDA-D-[c(RGDfK)]_2_ and ^201^Tl intravenously (IV), immediately followed by 20-min computed tomography (CT) scans (Fig. 1B). ^18^F-FDG PET/CT was conducted with the same animals at the day of SPECT/CT imaging performed. The PET images were obtained for 90 minutes with IV injection of ^18^F-FDG after CT scans for 20 minutes (Fig. 1C).

### SPECT Imaging of Viable Myocardium with ^201^Tl and ^99m^Tc-IDA-D-[c(RGDfK)]_2_

**Figure 2:**
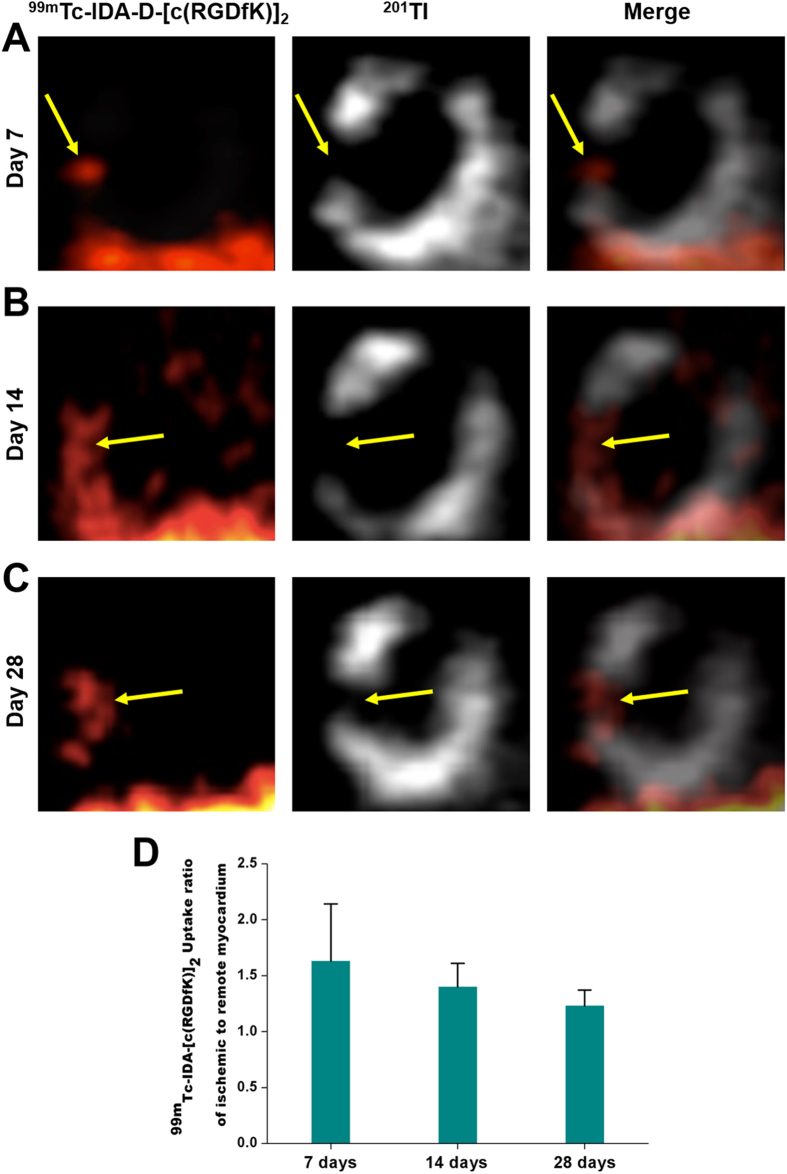
*In vivo* SPECT imaging of MI/reperfusion model with ^201^Tl and ^99m^Tc-IDA-D-[c(RGDfK)]_2_. (**A–C**) Serial SPECT scans were acquired at 7 (**A**), 14 (**B**), and 28 (**C**) days after MI/reperfusion. Shown are representative vertical long axis images from four independent experiments (n = 4 rats). The transiently ischemic, but still viable myocardium (arrows) was noted by strong signal of ^99m^Tc-IDA-D-[c(RGDfK)]_2_ (left column) and defect of perfusion signal, ^201^Tl (middle column). Images from two energy windows (^99m^Tc-IDA-D-[c(RGDfK)]_2_: 130–150 keV, ^201^Tl: 60–90 keV) were merged (right column) to identify localization of dual isotopes. (**D**) ^99m^Tc-IDA-D-[c(RGDfK)]_2_ uptake ratio of ischemic to remote myocardium was peak at day 7 (1.63 ± 0.51) and gradually decreased, but high enough for clear differentiation even 28 days later. Data are means ± SD (n = 4).

Using a radiolabeling protocol similar to that is described in our previous reports^21^,^22^, we produced ^99m^Tc-IDA-D-[c(RGDfK)]_2_, a diagnostic imaging agent for angiogenesis, with chemical and radiochemical purities greater than 99% and specific activity greater than 55 GBq/μmol. This agent, an integrin-binding RGD dimer peptide was designed to have increased hydrophilicity for optimized *in vivo* imaging. Its superior pharmacokinetic properties and high metabolic stability have been verified in the previous studies^21^,^22^. Herein we demonstrated the feasibility of SPECT imaging using ^99m^Tc-IDA-D-[c(RGDfK)]_2_ to noninvasively detect hibernating (i.e., ischemic but viable) myocardium in surgically generated rat models of transient coronary occlusion. Figure 2 presents representative vertical long axis images of MI models received with ^201^Tl and ^99m^Tc-IDA-D-[c(RGDfK)]_2_ at different time points after coronary occlusion. Images from two energy windows (^99m^Tc-IDA-D-[c(RGDfK)]_2_: 130–150 keV, ^201^Tl: 60–90 keV) were merged to identify localization of dual isotopes. High local uptake of ^99m^Tc-IDA-D-[c(RGDfK)]_2_ was detected in the ischemic region (i.e., left ventricular myocardium) with myocardial perfusion defect showing reduced signal of ^201^Tl at 7, 14, and 28 days after MI/reperfusion (Fig. 2A–C). Quantification of ^99m^Tc-IDA-D-[c(RGDfK)]_2_ uptake ratio of ischemic to remote myocardium using standardized uptake value (SUV) showed that the value was peak at day 7 (1.63 ± 0.51) and gradually decreased in process of time, but high enough for clear differentiation between ischemic and normal regions even at day 14 (1.4 ± 0.21) and day 28 (1.23 ± 0.14) (Fig. 2D). Nonspecific uptake of ^99m^Tc-IDA-D-[c(RGDfK)]_2_ in the liver was identified by strong SPECT signal at the bottom in Fig. 2A–C, but it does not interfere myocardial signals.

### ^18^F-FDG PET Imaging of Viable Myocardium in MI/reperfusion Model

**Figure 3:**
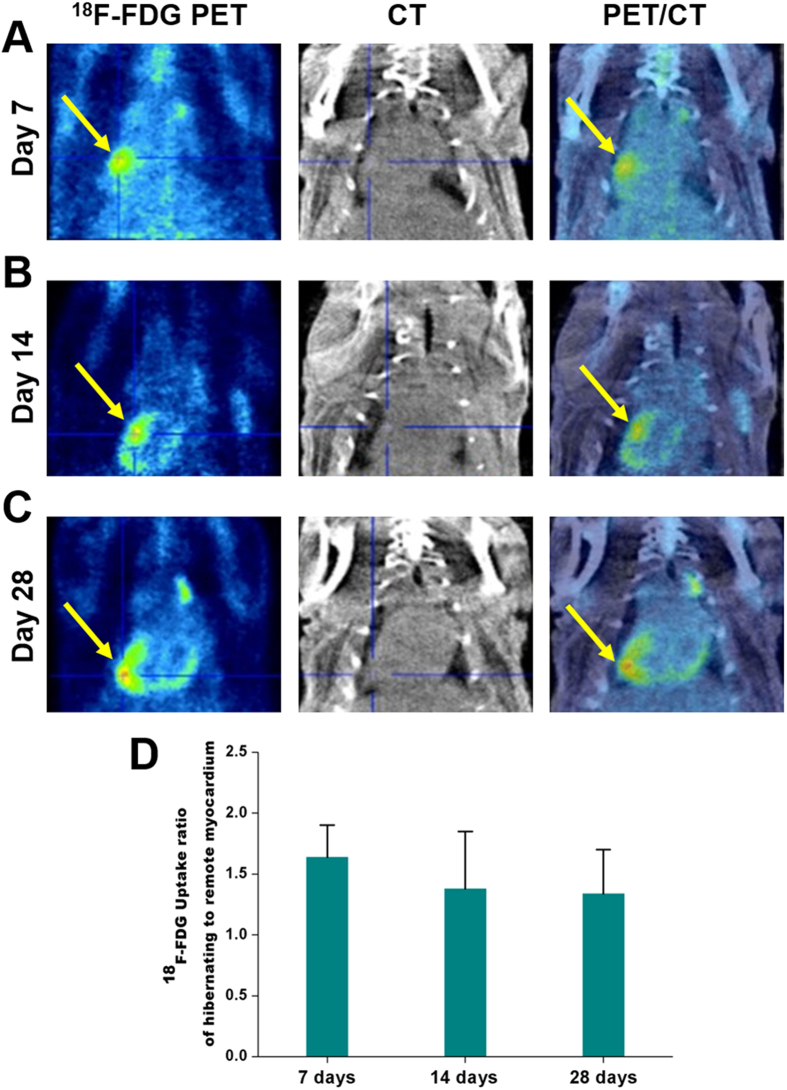
*In vivo* PET imaging of MI/reperfusion model with ^18^F-FDG. (**A–C**) Serial PET scans were acquired at 7 (**A**), 14 (**B**) and 28 (**C**) days after MI/reperfusion. Shown are representative vertical long axis PET (left column), CT (middle column) and PET/CT fusion (right column) images from four independent experiments (n = 4 rats). ^18^F-FDG PET/CT was conducted with the identical animal at the same day of ^99m^Tc-IDA-D-[c(RGDfK)]_2_ SPECT/CT imaging. The focal uptake of ^18^F-FDG (arrows) was seen in the identical region delineated by marked signal of ^99m^Tc-IDA-D-[c(RGDfK)]_2_ in SPECT imaging. (**D**) ^18^F-FDG uptake ratio of hibernating to remote myocardium was peak at day 7 (1.64 ± 0.26) and gradually decreased similarly with ^99m^Tc-IDA-D-[c(RGDfK)]_2_ SPECT imaging. Data are means ± SD (n = 4).

‘Perfusion defect’ identified by loss of ^201^Tl signal means the presence of scar (i.e., irreversible damage) or hibernating (i.e., reversible damage) myocardium^2^,^4^. To clarify whether intense uptake of ^99m^Tc-IDA-D-[c(RGDfK)]_2_ would come from fibrous scar (non-viable myocardium) or viable myocardium, we next performed glucose metabolic imaging with ^18^F-FDG PET by using the same rats on the day of SPECT imaging. As shown in Fig. 3, the focal uptake of ^18^F-FDG was observed in the ischemic region associated with marked signal of α_v_β_3_ integrin-targeting radiotracer ^99m^Tc-IDA-D-[c(RGDfK)]_2_ and reduced perfusion assessed with ^201^Tl tomographic imaging. ^18^F-FDG uptake in the normal remote myocardium was minimized under fasting condition which is current clinical protocol of myocardial PET with ^18^F-FDG. Evaluation for uptake ratio of hibernating to remote myocardium with ^18^F-FDG revealed time course variation similar to that with ^99m^Tc-IDA-D-[c(RGDfK)]_2_, showing peak at day 7 (1.64 ± 0.26) and gradual decrease at day 14 (1.38 ± 0.47) and day 28 (1.34 ± 0.36) (Fig. 3D). Currently, the American Society of Nuclear Cardiology (ASNC) guidelines^23^,^24^ recommend the use of perfusion together with ^18^F-FDG to assist in defining the region where perfusion is reduced whereas metabolism is preserved, the hallmark of hibernation and the parameter most related to patient risk for adverse outcomes if they do not receive appropriate therapies such as coronary revascularization. Thus, preferential *in vivo* accumulation of ^99m^Tc-IDA-D-[c(RGDfK)]_2_ in the region having perfusion-metabolism mismatch pattern indicated that the developed agent has good specificity for delineating myocardial hibernation.

### Correlation Analysis of ^18^F-FDG PET and ^99m^Tc-IDA-D-[c(RGDfK)]_2_ SPECT Scans

**Figure 4:**
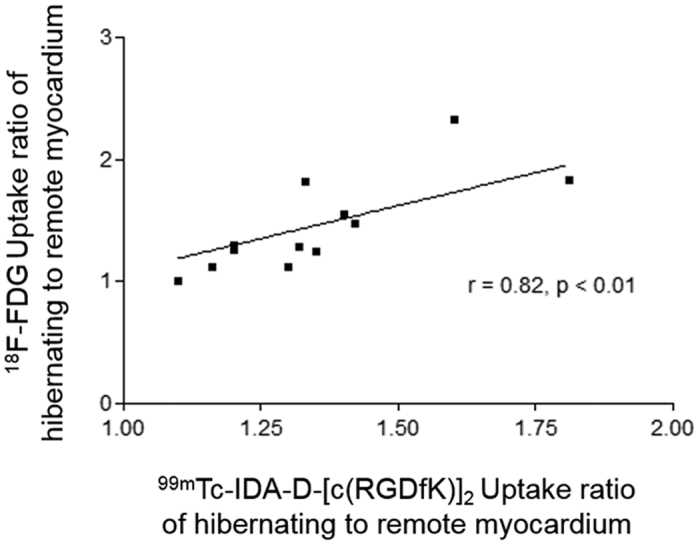
Correlation of radiotracer uptake ratio of hibernating to remote myocardium between ^18^F-FDG PET and ^99m^Tc-IDA-D-[c(RGDfK)]_2_ SPECT. Quantification and statistical analysis revealed a good linear relationship (*r* = 0.82, *p* < 0.01) between ^18^F-FDG PET and ^99m^Tc-IDA-D-[c(RGDfK)]_2_ SPECT data. Correlation coefficient was calculated using Spearman’s rank correlation.

At present, ^18^F-FDG PET imaging is considered to be the most sensitive means to measure myocardial viability for predicting improvement in left ventricular function after revascularization^25^. To confirm that angiogenesis targeting ^99m^Tc-IDA-D-[c(RGDfK)]_2_ SPECT can be a surrogate tool of ^18^F-FDG PET, we conducted correlation analysis between uptake ratios of hibernating to remote myocardium of ^99m^Tc-IDA-D-[c(RGDfK)]_2_ and ^18^F-FDG. *In vivo* time course images of ^99m^Tc-IDA-D-[c(RGDfK)]_2_ (n = 4) and ^18^F-FDG (n = 4) showed comparably high specific uptake of both tracers in the transiently occluded myocardium. SUV ratio evaluation of ^99m^Tc-IDA-D-[c(RGDfK)]_2_ and ^18^F-FDG imaging in MI models also revealed similar uptake in hibernating myocardium. As a result, a strong correlation between ^99m^Tc-IDA-D-[c(RGDfK)]_2_ and ^18^F-FDG was found by statistical analysis (*r* = 0.82, *p* < 0.01 by Spearman’s rank correlation analysis, Fig. 4), indicating the noninferiority of ^99m^Tc-IDA-D-[c(RGDfK)]_2_ as compared to ^18^F-FDG.

### Immunohistochemical Characterization of ^99m^Tc-IDA-D-[c(RGDfK)]_2_ High Myocardium

**Figure 5:**
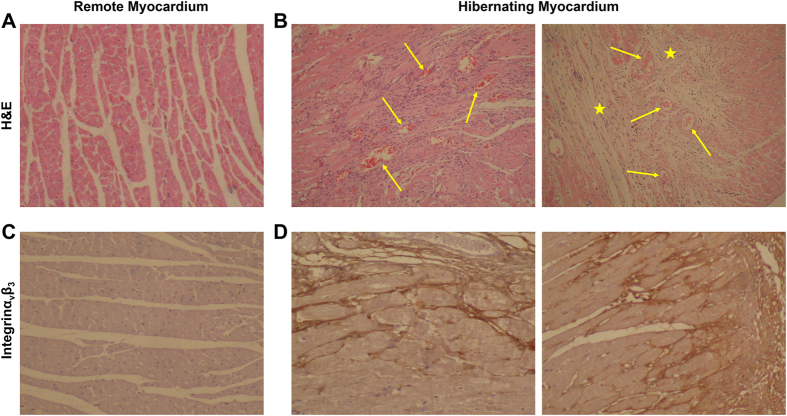
Immunohistochemical characterization of myocardium in MI/reperfusion model. Shown are representative photomicrographs of histologically stained cross sections of cardiac tissue isolated from MI/reperfusion model after *in vivo* SPECT and PET imaging. (**A,B**) Hematoxylin and eosin stained images display dense and aligned muscle structure in the remote myocardium (**A**) whereas loose and fibrous characteristics (asterisks) with abundance of micro-vessels (arrows) in the hibernating myocardium (**B**). (**C,D**) Immunohistochemical staining shows preferential expression of activated endothelial cell marker Integrin α_v_β_3_ (brown color) in the hibernating myocardium, indicative of angiogenesis-rich characteristics.

After *in vivo* SPECT imaging, we then histopathologically characterized myocardial specimens with high and low uptake of ^99m^Tc-IDA-D-[c(RGDfK)]_2_, corresponding to hibernating and remote regions (Fig. 5). ^99m^Tc-IDA-D-[c(RGDfK)]_2_ uptake was the highest in the specimens with morphologically loose and fibrous characteristics with abundance of micro-vessels (as shown by Hematoxylin and eosin staining, Fig. 5B) and correlated with the extent of α_v_β_3_ integrin activation (as shown by immunohistochemical staining with monoclonal antibody to α_v_β_3_ integrin, Fig. 5D). On the other hand, myocardium with low signal of ^99m^Tc-IDA-D-[c(RGDfK)]_2_ showed dense and aligned muscle structure, scarce presence of micro-vessels, and lack of α_v_β_3_ integrin expression. Pathological analysis revealed significant correlation between *in vivo* uptake of ^99m^Tc-IDA-D-[c(RGDfK)]_2_ and their respective characteristics of angiogenesis rich-viable myocardium.

## Discussion

Despite considerable diagnostic and therapeutic advances for ischemic cardiovascular disease (CVD) over the past 40 years^26^,^27^,^28^,^29^, there remains a significant population of patients who are not managed well by current treatment approaches^1^. One of the main reason for this failure is clinical challenge to assess individual risk factor. The identification of a so-called ‘hibernating myocardium’ is the most important in predicting which patients will experience functional recovery after revascularization and which patients will not. Although perfusion and metabolic imaging have been widely used for diagnosing CVDs, such strategies do not reflect the activity of key biomarkers identifying myocardial viability such as angiogenesis. Another reason resulting in high mortality of CVD patients is the absence of novel revascularization therapy capable of sufficient cardiac repair. Angiogenic therapy is an attractive approach for regeneration of ischemic myocardium, however, as yet none have shown sufficient efficacy to be approved in clinical trials^7^,^30^,^31^. As more and more specific therapies emerge with a variety of angiogenic agents^5^,^6^, the need for equally specific diagnostic tests is growing to lead us into clear success story. Accordingly, there is an unmet clinical need to develop a specific imaging tool for evaluation of myocardial salvage, suitable candidate selection, and efficacy monitoring of novel therapy by targeting angiogenesis and angiogenesis rich-viable myocardium.

In this study, we showed the feasibility of SPECT imaging with α_v_β_3_ integrin-targeted ^99m^Tc-IDA-D-[c(RGDfK)]_2_ for specific detection of angiogenesis rich-viable myocardium in a rat model of transient coronary occlusion. Having motivated by our recent findings that ^99m^Tc-IDA-D-[c(RGDfK)]_2_ SPECT can sensitively detect angiogenesis in tumor tissue and atherosclerotic plaque, we further explored the potential of this radiotracer for identification of angiogenesis rich-viable myocardium which is important for myocardial infarction staging and angiogenesis associated risk stratification. *In vivo* SPECT imaging showed focal increase of the ^99m^Tc-IDA-D-[c(RGDfK)]_2_ signal in the region with reversible myocardial injury by 20-min coronary artery occlusion. In particular, we found such an intense uptake of ^99m^Tc-IDA-D-[c(RGDfK)]_2_ corresponds to perfusion-metabolism mismatch pattern (i.e., signature of hibernation) identified by reduced ^201^Tl SPECT and enhanced ^18^F-FDG PET signals. The uptake of ^99m^Tc-IDA-D-[c(RGDfK)]_2_ was non-inferior to ^18^F-FDG uptake, confirmed by linear relationship in time course variation analysis. Histopathological characterization corroborated the *in vivo* data by revealing abundance of micro-vessels and elevated expression of α_v_β_3_ integrin in ^99m^Tc-IDA-D-[c(RGDfK)]_2_ signal high myocardium. These data indicated that ^99m^Tc-IDA-D-[c(RGDfK)]_2_ SPECT may serve as a promising clinical measure for prediction of revascularization efficacy of the individual patients.

Current revascularization procedures aimed at reopening the obstructed artery, such as thrombolysis, angioplasty, and bypass surgery improved the post-MI survival rate. To date, perfusion and metabolism imaging have become the gold standard for diagnosis, prognosis, and follow-up of the patients treated by revascularization. In contrast, specific molecular imaging has not yet been used extensively to characterize injured myocardium although the quest for new, potentially more specific targeting strategies has continued. Angiogenesis is a key factor in the process of cardiac healing after myocardial ischemia. As a result, much attention is paid to targeting angiogenesis for both diagnosis and therapy. However, molecular imaging strategy to track angiogenesis never been rigorously evaluated by comparison with current clinical standard, i.e., the combined measurement of myocardial perfusion and metabolism. The main advance of this work comes from this point. We clarified that angiogenesis targeting with the developed SPECT agent ^99m^Tc-IDA-D-[c(RGDfK)]_2_ can exactly pinpoint injured but viable myocardium coincident with current diagnostic criteria (i.e., perfusion defect and metabolic activation). This was possible by the use of a carefully designed and validated transient coronary occlusion model. The demonstration of comparable imaging performance to assess myocardial viability in comparison with gold standard approach will facilitate clinical translation of ^99m^Tc-IDA-D-[c(RGDfK)]_2_ based angiogenesis imaging technique to manage CVDs.

Much work is still ahead to clinically apply ^99m^Tc-IDA-D-[c(RGDfK)]_2_ SPECT for the use in ischemic heart disease to enable reliable detection of hibernation, offering information on the likelihood of response to a given therapy. Of note, an urgent need exists to investigate the capability of the α_v_β_3_ integrin targeted ^99m^Tc-IDA-D-[c(RGDfK)]_2_ to capture multiple components to boost cardiac recovery. Wound healing after MI entails a cascade of events including angiogenesis, inflammation and a complex interplay between several different cell types such as macrophages and myofibroblasts. Interestingly, α_v_β_3_ integrin is highly expressed not only in endothelial cells during angiogenesis but also in macrophages^32^,^33^ and myofibroblasts^12^,^34^. As such, it is worth to explore the specific range of *in vivo* targeting by the developed radiotracer. The strong correlation between ^99m^Tc-IDA-D-[c(RGDfK)]_2_ and ^18^F-FDG (Fig. 4) indicates that ^99m^Tc-IDA-D-[c(RGDfK)]_2_ SPECT can record not only angiogenesis but also macrophage activity because ^18^F-FDG is usually accumulated not in cardiac muscle cells but in activated macrophages under fasting condition. In addition, further work needs to be accomplished to clarify the complex relationship between the timing of imaging, signal strength, and functional outcome. Comparison with other suggested imaging strategy such as ^201^Tl SPECT with glucose-insulin-potassium (GIK) infusion will be also valuable to more accurately verify hibernating myocardium targeting ability of ^99m^Tc-IDA-D-[c(RGDfK)]_2_ SPECT. The potential of ^99m^Tc-IDA-D-[c(RGDfK)]_2_ SPECT imaging to distinguish delicate differences amongst different ischemic myocardium must be evaluated especially in order to clearly pinpoint hibernating myocardium from irreversible scar using different degree of occlusion models. To confirm whether ^99m^Tc-IDA-D-[c(RGDfK)]_2_ SPECT signal high region after myocardial injury, indicative of hibernating myocardium, could really show successful improvement in contractile function after revascularization will be of another great interest. Such assessment can be performed by physiological measurement on the regional left ventricular function using echocardiography or electrocardiography. Generalized, large, and prospective clinical trials are mandatory to reach beyond the tools available in laboratory research. Most importantly, however, this current study had laid the foundation for possible application of ^99m^Tc-IDA-D-[c(RGDfK)]_2_ from fundamental research of cardiac healing process to patient management and new revascularization drug development.

In summary, the presented study suggests that noninvasive identification of viable myocardium under ischemic heart condition is feasible using α_v_β_3_ integrin-targeted ^99m^Tc-IDA-D-[c(RGDfK)]_2_ which can be readily incorporated into clinical practice to identify the patients who might benefit most from revascularization. As a sensitive angiogenesis detection probe to monitor revascularization therapy effect, this imaging platform can also pave the way to the successful development of a drug capable of revascularization of cardiac tissues which would be a major milestone in the history of cardiovascular medicine.

## Materials and Methods

### Animal Model of Myocardial Infarction and Reperfusion

All animal experiments were carried out in accordance with the approved guidelines. All animal experimental protocols were approved by the Institutional Animal Care and Use Committee of Preclinical Research Institute in the Seoul National University Bundang Hospital. A total of eight Sprague-Dawley rats (280–400 g, male, Charles River Laboratories, Wilmington, MA, USA) were used for this study. Generation of transient coronary occlusion (MI) followed by reperfusion (n = 4) has been fully described by previous investigators^10^,^16^,^35^. Briefly, rats were anaesthetized with intraperitoneal administration of zoletil (30 mg/kg), xylazine (5 mg/kg) and then intubated and mechanically ventilated with a mixture of O_2_ and 1.5% isoflurane using a rodent ventilator (Harvard model 683, Harvard Apparatus Inc., Boston, MA, USA). Animals were placed in a supine position and the body temperature monitored and maintained at 35–37 °C. A left thoracotomy was then performed through the third intercostal space by a horizontal incision of pectoralis muscles to expose the heart. The anterior descending branch of the left coronary artery (LCA) was ligated using a 5–0 polypropylene suture with a small curved needle. The coronary occlusion was confirmed by the regional appearance of pale color on the anterior surface of the left ventricle (LV). Twenty minutes after the ligation, the suture was cut and removed to achieve reperfusion. The model of transient 20 min coronary occlusion was selected to induce MI in approximately half of the region at risk and generate ischemic but viable “hibernating myocardium” as previously reported^10^. The chest was closed, and then the animal was gradually weaned from the respirator as soon as they are able to breathe spontaneously. Additionally, control sham-operated rats underwent the same surgical procedures except the occlusion of the LCA (n = 4).

### Chemistry and Radiochemistry

All commercial reagents and solvents were purchased from Sigma-Aldrich (St. Louis, MO, USA) and used without further purification, unless otherwise specified. The precursor, IDA-D-[c(RGDfK)]_2_ was kindly provided by Bio Imaging Korea Co., Ltd. (Seoul, Republic of Korea), and the radiotracer, ^99m^Tc-IDA-D-[c(RGDfK)]_2_ was synthesized as described in the previous study^21^. ^99m^Tc-pertachnate was eluted on a daily basis from ^99^Mo/^99m^Tc-generator (Samyoung Unitech, Seoul, Republic of Korea) and ^18^F-Fluoride was produced by ^18^O(p, n)^18^F reaction through proton irradiation using a KOTRON-13 cyclotron (Samyoung Unitech, Seoul, Republic of Korea) at the Seoul National University Bundang Hospital. ^201^Tl was purchased from the Korea Institute of Radiological & Medical Sciences (Seoul, Republic of Korea).

### *In Vivo* SPECT/CT Imaging

Dual isotope SPECT/CT imaging with ^201^Tl and ^99m^Tc-IDA-D-[c(RGDfK)]_2_ was performed on MI/reperfusion models (n = 4) and sham-operated groups (n = 4). Serial SPECT/CT scans were acquired at 7, 14, and 28 days after MI/reperfusion for each identical animal. Experimental rats were anesthetized with 1.5–2% isoflurane in 100% oxygen (2 L/min flow rate). They were then placed supine on the bed of an animal SPECT/CT scanner (NanoSPECT/CT, Bioscan Inc., Washington DC, USA) and a bolus of ^99m^Tc-IDA-D-[c(RGDfK)]_2_ (1.20 ± 0.1 mCi) was injected intravenously. Ten minutes after injection of ^99m^Tc-IDA-D-[c(RGDfK)]_2_, ^201^Tl (0.78 ± 0.06 mCi) for evaluation of myocardial perfusion was given by intravenous injection. The data acquisition was initiated 20 min after ^99m^Tc-IDA-D-[c(RGDfK)]_2_ injection. SPECT imaging was performed using a low-energy and high-resolution pyramid collimator. Whole body images were obtained in 24 projections over a 30 min period using a 4-head scanner with 4 × 9 (1.4 mm) pinhole collimators in helical scanning mode. Images were acquired using two energy windows (^99m^Tc-IDA-D-[c(RGDfK)]_2_: 130–150 keV, ^201^Tl: 60–90 keV) and grouped into a single image.

SPECT imaging was followed by CT scans with the animal exactly in the same position. The animal CT scanner system consisting of a low-energy X-ray tube and a precision motion translation stage was used. The X-ray source and detectors are mounted on a circular gantry allowing it to rotate 360° around the rat positioned on a stationary bed. The images at 180 projections were acquired with the X-ray source set at 45 kVp and 177 μA. Two-dimensional slices of the bed position were reconstructed using an Exact Cone Beam Filter Back Projection algorithm with a Shepp-Logan filter. Finally, the CT image was used to correct attenuation error of the gamma-ray signal emitted from the ^201^Tl or ^99m^Tc-IDA-D-[c(RGDfK)]_2_.

### *In Vivo* PET/CT Imaging

In addition to the SPECT/CT, a PET/CT scan with ^18^F-FDG was done at the same day. All animals were fasted for 24 hours before imaging to follow the protocol of current clinical practice^36^, thus minimize ^18^F-FDG uptake in normal myocardium. Rats were anesthetized with 1.5–2% isoflurane in 100% oxygen (2 L/min flow rate). Following a 20-min CT scan for attenuation correction, the PET data acquisition was started at the time of intravenous injection with ^18^F-FDG (1.20 ± 0.09 mCi). The 3D static images were collected for 90 minutes with an energy window of 400–600 keV.

All PET scans were performed using a small animal PET scanner, NanoPET/CT (Bioscan Inc, USA), which provides a minimum axial coverage of 9.48 cm, a 12.3 mm transaxial field of view, 0.3 mm sampling distance, 0.58 mm in-plane reconstructed resolution and 7.7% of absolute sensitivity at the center of field of view for an energy window at 250–750 keV.

### Image Analysis

The acquired images were processed with the comprehensive image analysis software, PMOD (version 3.13, PMOD Technologies, Inc.). All emission images were co-registered with regard to the respective CT scan. The images (^99m^Tc-IDA-D-[c(RGDfK)]_2_ SPECT, ^201^Tl SPECT, and ^18^F-FDG PET) were then analyzed to calculate the standardized uptake values (SUVs) for a given subject at different time points. SUV at time point *t* is defined as follows:


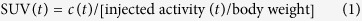


where c is the measured tissue radioactivity concentration (mCi/mL) and injected activity is the amount of radiation (mCi/mL) injected extrapolated to time point t. Three-dimensional volumes of interest (VOI) were drawn based on the radioactivity normalized SPECT or PET images. Subsequently, the mean SUV in each VOI of hibernating and remote myocardium was calculated. The uptake ratio of hibernating to remote zone for ^99m^Tc-IDA-D-[c(RGDfK)]_2_ and ^18^F-FDG was independently calculated by division of the mean SUV of hibernating VOI with that of remote VOI.

### Statistical Analysis

All quantitative data are expressed as mean ± SD. The correlation between quantitative parameters was evaluated by Spearman’s rank correlation. Statistical significance was tested using ANOVA. Differences with a *p* value less than 0.01 were considered statistically significant. All analyses were conducted with SPSS 15.0 statistical package (SPSS, Chicago, IL, USA) or MedCalc (MedCalc version 6.15.000).

### Histology and Immunohistochemistry

Following euthanasia of the rats underwent *in vivo* SPECT/CT and PET/CT imaging, myocardial tissue was isolated to confirm angiogenesis and integrin expression in the hibernating region by histopathological analysis. The excised tissues were fixed with 10% formalin, embedded in paraffin, cut into 5-μm sections and deparaffinized. The sections were then stained with hematoxylin and eosin or anti integrin α_ν_β_3_ monoclonal antibody (1:50, Abcam, ab7166) to identify characteristics of the recorded maximum and minimum radioactivity corresponding to the hibernating and remote myocardium, respectively. Bright field color micrographs were obtained on a BX51 microscope equipped with DP71 camera (Olympus Optical Co., Ltd., Tokyo, Japan).

## Additional Information

**How to cite this article**: Lee, M. S. *et al*. Identification of Angiogenesis Rich-Viable Myocardium using RGD Dimer based SPECT after Myocardial Infarction. *Sci. Rep.* **6**, 27520; doi: 10.1038/srep27520 (2016).
